# Genetic variation rs1121980 in the fat mass and obesity-associated gene (*FTO*) is associated with dietary intake in Koreans

**DOI:** 10.29219/fnr.v66.8059

**Published:** 2022-11-16

**Authors:** Young Goh, Jeong-Hwa Choi

**Affiliations:** Department of Food Science and Nutrition, Keimyung University, Daegu, Korea

**Keywords:** food intake, food preference, FTO, genetic variation, Korean, obesity

## Abstract

**Background:**

Fat mass and obesity-associated gene (*FTO*) is a well-known gene associated with body weight and obesity risk. Recent studies have suggested that genetic variations in *FTO* may play a role in the regulation of food preference and consumption. However, little is known with respect to Asian populations.

**Objective:**

This study examined whether rs1121980 C > T in *FTO* is associated with food intake in Koreans.

**Design:**

This study was performed using data from the Korean Genome and Epidemiology Study (Ansan/Ansung cohort). Dietary intake was determined using the semi-food frequency questionnaire, and the *FTO* rs1121980 genotypes of 6,262 individuals (3,049 males and 3,213 females) were analyzed along with sex and body mass index (BMI).

**Result:**

Genetic variation did not show a significant association with the population’s energy-nutrient intake. However, female T allele carriers with BMI ≥ 25 consumed more blue fish and coffee, and their coffee creamer consumption was decisively higher than that of T allele non-carriers (*P*_adjusted_ = 0.004). In males, the presence of the T allele showed a putative association with the consumption of sweets, snacks, and coffee creamer by the BMI level.

**Conclusion:**

The *FTO* rs1121980 variation was associated with a preference for foods particularly high in fat (e.g. coffee creamer, blue fish, sweets, and snacks) in Koreans; these preferences varied by sex and BMI.

## Popular scientific summary

- This study examined the association between the genetic variation rs1121980 in *FTO* and dietary and nutritional intakes in a large-scale epidemiological study cohort of Koreans.
- The *FTO* rs1121980 T obesity risk allele was associated with fat-tasting food intake, including coffee creamer, in Koreans.
- *FTO* genetic variation had no significant effect on macronutrient intake in Koreans.

Obesity is a disease in which fat accumulates in fat tissues in the body (1), and such excess body fat can negatively affect health. In 2018, the prevalence of obesity among Korean adults was 45.4% among males and 26.5% among females (2). Moreover, the prevalence of abdominal obesity in Korea has increased over the past decade, especially among males (28.1%) (2). Obesity increases the risk of developing type 2 diabetes, cardiovascular diseases, metabolic syndrome, and cancers (3). Obesity is also known to be related to psychological conditions, such as depression, anorexia, and bulimia (4).

Genetic variants have an important role in adiposity including in the number of adipocytes, the distribution of body fat, obesity development, and energy consumption (1). Earlier genome-wide association studies reported that single nucleotide polymorphisms (SNPs) present in the first intron of the fat mass and obesity-associated gene (*FTO*) increase the risk of obesity by 1.2-fold and are associated with an average increase in body mass index (BMI) of 0.39 kg/m^2^ (body weight 1,130 g) (5). For instance, SNPs in the *FTO* gene (rs178117449, rs8050136, rs1421085, rs9930506, rs9939609, and rs1121980) are associated with weight, BMI, body composition, and obesity risk. Such an association has been commonly evident in multiple age and ethnic groups (5).

The precise role of *FTO* in obesity etiology has not yet been revealed. However, among the potential mechanisms of action, *FTO* may be associated with controlling dietary intake, and this may possibly contribute to *FTO*-related disease risk: *FTO* is highly expressed in the hypothalamus, a region involved in appetite regulation. People with a higher risk of obesity had higher levels of ghrelin, an appetite-promoting hormone, than those with a lower risk of obesity and showed a strong appetite or preference (6). Earlier studies have provided evidence that *FTO* genetic variation may play a role in the regulation of food intake and preference (7, 8). In a study of 4,839 subjects from the Swedish Malmö Diet and Cancer study (MDCS) cohort, rs9939609 of the *FTO* gene showed a significant association with obesity risk related to fat and carbohydrate intake (7). Another study in the same cohort also reported that *FTO* genetic variation was associated with certain food intake, especially energy-dense foods (8). In the case of *FTO* rs1121980, homozygous subjects for the obesity risk allele had a higher saturated fat intake in Americans, but no significant association with carbohydrate intake was evident (9). However, little is known with respect to Asian populations. Since each race/ethnicity has its own physiological metabolism, eating habits, and cultures, the effects of genetic variants may vary. Therefore, we evaluated whether *FTO* rs1121980 is associated with dietary intake in Koreans. This study was performed to ascertain the association of the *FTO* rs1121980 genetic variation with the intake of macronutrients and selected food groups in Koreans stratified by the BMI level. Additionally, the study employed a sex-stratified approach since sex differences exist in health and dietary behaviors (10).

## Materials and methods

### Study subjects and data collection

**Fig. 1:**
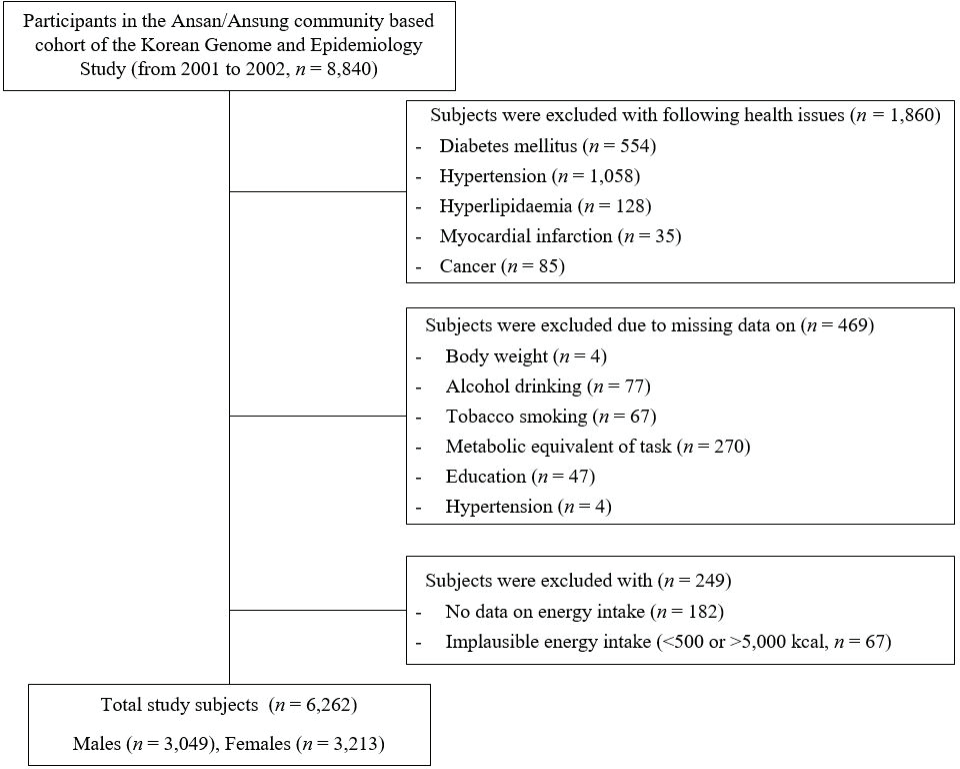
Simplified process of study subjects selection. *n*, number of subjects.

This study was conducted with data from a population-based cohort, the Ansan and Ansung study, a part of the Korean Genome and Epidemiology Study (KoGES) conducted from 2001 to 2002. The KoGES is a cohort project initiated by the Korea Centers for Disease Control and Prevention to identify the relationship between risk factors and chronic diseases that commonly affect Koreans. Subjects were 40- to 69-year-old adults living in Ansan (urban) and Ansung (rural) in Gyeonggi Province (11). Of the 8,840 subjects with genetic and epidemiological characteristic data, subjects with a history of diabetes (*n* = 554), hyperlipidemia (*n* = 128), myocardial infarction (*n* = 35), or cancer (*n* = 85) and no information or history of hypertension (*n* = 1,062) were excluded. Additionally, subjects without anthropometric (*n* = 4), alcohol consumption (*n* = 77), or smoking (*n* = 67), physical activity (*n* = 270), and education level (*n* = 47) information were also excluded from the dataset. Finally, this study was conducted with 3,049 males and 3,213 females, for a total of 6,262 subjects, excluding those with unknown daily energy intake (*n* = 182) or implausible total caloric intake (<500 or >5,000 kcal/day, *n* = 67, Fig. 1). The KoGES was conducted following a protocol approved by the Institutional Review Board (IRB). All subjects provided written consent before study initiation. This study was approved by the IRB (40525-201802-HR-121-07).

### Collection of general characteristics and anthropometric measures

General characteristics such as sex, region, age, and educational level of KoGES study subjects were collected through a baseline survey (11). According to smoking and alcohol consumption, subjects were classified into three groups: never, past, and current. The level of education was classified into three groups: low (elementary school or less), middle (middle or high school), and high (college or higher). The intensity of each type of physical activity was evaluated by calculating the metabolic equivalent of task (MET) using the type and duration of physical activity (12). The BMI of the subjects was computed as weight (kg) divided by the squared height (m^2^).

### Collection and analyses of dietary data

Using semi-quantitative food frequency questionnaire (SQFFQ) data collected by the KoGES, the subjects’ diet and nutrition conditions were investigated (13). Subjects were interviewed on the amount and frequency of 103 food items they ate over the past year. To investigate the influence of *FTO* rs1121980 on Koreans’ food intake, among the 103 foods, selected items were grouped as follows by taking into account Koreans’ dietary culture and earlier studies (8): all vegetables, all fruits, meats, white meats, red meats, organ meats, fishes, blue fishes, white fishes, sweets and snacks, coffee, and coffee creamer. Additionally, there were nine options for the frequency of intake: never or barely, 1 time a month, 2~3 times a month, 1~2 times a week, 3~4 times a week, 5~6 times a week, 1 time a day, 2 times a day, or ≥3 times a day. The serving size of each food item consists of three options: small, medium, or large. Food intake was converted to daily average nutritional intake using the database of The Korean Nutrition Society (14).

### Genotyping and SNP selection

DNA samples for genotype analysis were obtained from the subject’s peripheral blood. Detailed information is described elsewhere (15). Briefly, the genotype was analyzed using Affymetrix Genome-Wide Human SNP array 5.0 (Affymetrix Inc., Santa Clara, CA, USA), and quality control of data was performed under Bayesian robust linear modelling using the Mahalanobis distance algorithm. Samples were excluded if they presented low quality, including sex and ethnic inconsistencies, genotyping call rate <96%, excessive heterozygosity, or cryptic relatedness. Genetic loci were excluded if they possessed a call rate <95%, had a low minor allele frequency (MAF) of <0.01, or deviated from Hardy–Weinberg equilibrium (*P* < 1 × 10^-6^). From the KoGES dataset, three genetic variants (rs9939973, rs9939609, and rs1121980) in *FTO* region were available. According to the LDlink analyses, rs1121980 was strongly associated with the other two variants in the Asian population (*D*’ = 1.0 for both, *r* ^2^ = 0.78 and 1.0 for rs9939973 and rs9939609, respectively) (16). From the preliminary analyses, rs1121980 showed a similar but rational association with dietary intake than the two other variants. For this reason, further analyses were performed with the rs1121980 variation (Supplementary Tables 1–4).

### Statistical methods

General characteristics of the subjects were analyzed by applying Student’s *t*-test and Chi-squared test according to the types of variables. Continuous variables such as age, height, weight, BMI, METs, and food and nutrition intake were logarithmically transformed before analyses. Food and macronutrient intake data were analyzed after adjustment for total energy intake using Willett’s residual method (17). In the case of any food intake being zero, a small value (0.001) was added before logarithmic transformation (8).

Analyses were also performed in two BMI subgroups defined following the Korean Society for the Study of Obesity guidelines: normal weight and obese (BMI < 25 or ≥25 kg/m^2^) (18). General linear models were used to test the difference in dietary and nutrient intake according to *FTO* genotype. Model I is a crude statistical model. Model II is an adjusted model considering covariates such as residential area, age, BMI, educational level, alcohol consumption, smoking status, and physical activity.

Statistical analyses were performed using SAS version 9.4 (Statistical Analysis System, SAS Institute Inc., Cary, NC, USA) and SPSS version 26 (Statistical Package for Social Science, SPSS Inc., Chicago, IL, USA). Two-tailed *P*-values < 0.0042 were recognized as statistically significant to correct for multiple testing issues following Bonferroni’s rule (0.0042 = 0.05/12, number of dietary-related variables examined).

## Results

### General characteristics of study subjects by FTO rs1121980 genotype and BMI level

The descriptive data of the subjects according to *FTO* rs1121980 genotype and sex are shown in Table 1. A total of 6,262 individuals (male = 3,049; female = 3,213) with known *FTO* rs1121980 genotypes were analyzed using a sex-stratified approach. Among them, 2,166 males (71.0%) and 2,286 females (71.1%) had the CC genotype, while 83 males (2.7%) and 85 females (2.6%) had the TT genotype. Because of the limited number of subjects with the TT genotype, the subjects were grouped according to the presence of the T allele. Thus, 883 males (29.0%) and 927 females (28.9%) were defined as minor T allele carriers, and the following analyses were performed according to the presence of the T allele (CT + TT versus CC genotype).

**Table 1:** General characteristics of the study population by taking into account the FTO rs1121980 genotype and sex

Males (*n* = 3,049)	BMI < 25 (*n* = 1,915)			BMI ≥ 25 (*n* = 1,134)		
	CC (*n* = 1,375)	CT + TT (*n* = 540)	*P*	CC (*n* = 791)	CT + TT (*n* = 343)	*P*
Age (years)	51.7 ± 9.0	51.8 ± 9.0	0.885	49.5 ± 7.8	48.4 ± 7.1	0.028
Region			0.490			0.638
Rural (Ansung)	607 (72.6)	229 (27.4)		258 (70.7)	107 (29.3)	
Urban (Ansan)	768 (71.2)	311 (28.8)		533 (69.3)	236 (30.7)	
Height (cm)	166.8 ± 5.9	166.7 ± 5.9	0.809	167.6 ± 5.7	167.3 ± 5.5	0.437
Weight (kg)	62.0 ± 7.2	62.5 ± 6.9	0.161	76.0 ± 7.1	75.7 ± 6.8	0.517
BMI (kg/m^2^)	22.3 ± 1.9	22.5 ± 1.9	0.038	27.0 ± 1.6	27.0 ± 1.6	0.913
Physical activity, METs	25.7 ± 15.6	25.0 ± 15.5	0.222	22.0 ± 13.7	21.5 ± 12.9	0.577
Alcohol consumption			0.909			0.461
Non-drinker	258 (71.9)	101 (28.1)		135 (67.8)	64 (32.2)	
Ex-drinker	123 (73.2)	45 (26.8)		71 (65.7)	37 (34.3)	
Current drinker	994 (71.6)	394 (28.4)		585 (70.7)	242 (29.3)	
Smoking status			0.557			0.815
Non-smoker	245 (69.6)	107 (30.4)		163 (71.5)	65 (28.5)	
Ex-smoker	445 (72.8)	166 (27.2)		305 (69.3)	135 (30.7)	
Current smoker	685 (72.0)	267 (28.0)		323 (69.3)	143 (30.7)	
Education			0.462			0.702
High	276 (74.2)	96 (25.8)		197 (69.4)	87 (30.6)	
Middle	791 (70.9)	325 (29.1)		480 (69.3)	213 (30.7)	
Low	308 (72.1)	119 (27.9)		114 (72.6)	43 (27.4)	

Females (*n* = 3,213)	BMI < 25 (*n* = 1,921)			BMI ≥ 25 (*n* = 1,292)		
	CC (*n* = 1,384)	CT + TT (*n* = 537)	*P*	CC (*n* = 902)	CT + TT (*n* = 390)	*P*

Age (years)	50.7 ± 9.0	50.5 ± 9.1	0.702	52.1 ± 8.5	51.7 ± 8.5	0.485
Region			0.821			0.882
Rural (Ansung)	611 (72.3)	234 (27.7)		440 (69.6)	192 (30.4)	
Urban (Ansan)	773 (71.8)	303 (28.2)		462 (70.0)	198 (30.0)	
Height (cm)	154.4 ± 5.7	154.6 ± 5.6	0.551	153.4 ± 5.3	154.0 ± 5.5	0.099
Weight (kg)	53.7 ± 5.8	53.9 ± 5.7	0.398	65.0 ± 7.0	65.3 ± 7.2	0.448
BMI (kg/m^2^)	22.5 ± 1.8	22.5 ± 1.8	0.543	27.6 ± 2.2	27.5 ± 2.4	0.644
Physical activity, METs	22.3 ± 14.0	22.6 ± 14.2	0.470	23.3 ± 14.5	22.8 ± 14.1	0.923
Alcohol consumption			0.773			0.147
Non-drinker	972 (72.2)	375 (27.8)		636 (71.1)	258 (28.9)	
Ex-drinker	38 (76.0)	12 (24.0)		30 (75.0)	10 (25.0)	
Current drinker	374 (71.4)	150 (28.6)		236 (65.9)	122 (34.1)	
Smoking status			0.263			0.258
Non-smoker	1,328 (72.4)	506 (27.6)		850 (69.3)	376 (30.7)	
Ex-smoker	29 (64.4)	16 (35.6)		28 (80.0)	7 (20.0)	
Current smoker	27 (64.3)	15 (35.7)		24 (77.4)	7 (22.6)	
Education			0.235			0.976
High	113 (77.4)	33 (22.6)		46 (70.8)	19 (29.2)	
Middle	758 (70.9)	311 (29.1)		442 (69.9)	190 (30.1)	
Low	513 (72.7)	193 (27.3)		414 (69.6)	181 (30.4)	

BMI, body mass index; METs, metabolic equivalent of task; *n*, number of subjects.

Values are presented as the means and standard deviations for age, height, weight, body mass index, metabolic equivalent of task, and nutrients; the others are the numbers of subjects with percentages in parentheses.

*P*-values were from Student’s t-tests for age, height, weight, and BMI, otherwise Chi-squared tests.

Age, residential area, height, weight, physical activity, alcohol consumption, smoking status, and the level of education in subjects were not significantly different by the *FTO* rs1121980 genotype. In the case of BMI, there was a difference between normal male genotypes. T allele carriers (CT and TT genotypes) tended to have a higher BMI than CC carriers (22.5 ± 1.9 and 22.3 ± 1.9 kg/m^2^, *P* = 0.038). In females, neither the normal nor obese groups were associated with BMI.

### FTO rs1121980 genotype, total energy, and macronutrient intake

The results of the analysis of whether the *FTO* rs1121980 genotype, total energy, and macronutrient intake were associated are shown in Table 2. In males, both crude model I and adjusted model II suggested that the presence of the T allele was not associated with differences in the consumption of total energy, carbohydrate, fat, and protein. In the case of females, again, both models I and II suggested that the T allele showed no association with total energy and macronutrient intake.

**Table 2:** Level of macronutrient consumption and the association with FTO rs1121980 genotype in normal weight and overweight individuals

Macronutrient	BMI < 25				BMI ≥ 25			
	CC	CT + TT	*P*_crude_	*P*_adjusted_	CC	CT + TT	*P*_crude_	*P*_adjusted_
Males	*n* = 1,375	*n* = 540			*n* = 791	*n* = 343		
Total energy (kcal/day)	1,963.9 ± 572.2	1,983.4 ± 573.5	0.558	0.548	2,060.0 ± 582.9	2,052.4 ± 531.8	0.981	0.941
Carbohydrate (g/day)	341.4 ± 95.9	343.1 ± 93.2	0.731	0.721	353.8 ± 96.1	350.1 ± 84.8	0.718	0.757
Protein (g/day)	66.8 ± 24.6	67.8 ± 26.2	0.479	0.480	71.8 ± 25.6	71.6 ± 24.6	0.895	0.833
Fat (g/day)	34.3 ± 18.0	35.2 ± 19.2	0.407	0.370	37.3 ± 18.9	38.1 ± 17.2	0.300	0.436
Females	*n* = 1,384	*n* = 537			*n* = 902	*n* = 390		
Total energy (kcal/day)	1,859.2 ± 635.3	1,882.8 ± 602.9	0.240	0.225	1,890.9 ± 605.8	1,894.6 ± 636.5	0.900	0.781
Carbohydrate (g/day)	331.0 ± 112.6	334.3 ± 106.7	0.323	0.301	340.1 ± 106.9	339.0 ± 114.2	0.606	0.528
Protein (g/day)	62.9 ± 26.4	63.7 ± 23.7	0.217	0.207	63.6 ± 25.2	63.8 ± 25.4	0.981	0.932
Fat (g/day)	29.7 ± 18.2	30.4 ± 16.9	0.162	0.141	28.8 ± 16.7	29.6 ± 16.9	0.479	0.623

BMI, body mass index; *n*, number of subjects.

Values are presented as the means and standard deviations.

*P*_crudes_ were from crude general linear models.

*P*_adjusteds_ were from general linear models including region, age, body mass index, education level, alcohol consumption, smoking status, and physical activity level.

### FTO rs1121980 genotype and dietary intake

In further analyses, we evaluated the association between the *FTO* rs1121980 genetic variant and dietary consumption, taking into account obesity status and sex. Our findings suggested that obese males with *FTO* rs1121980 T alleles tended to consume more sweets and snacks; moreover, in the group of males with normal BMI, minor T allele carriers tended to consume more coffee creamer (Table 3). However, such diet and genotype associations were not significant in model II adjusted for covariates, including residential area, age, BMI, education level, alcohol consumption, smoking status, and physical activity METs.

**Table 3:** Consumption of selected foods (g/day) and the association with the FTO rs1121980 genotype in males

Food group	BMI < 25 (*n* = 1,915, 62.8%)				BMI ≥ 25 (*n* = 1,134, 37.2%)			
	CC (*n* = 1,375)	CT + TT (*n* = 540)	*P*_crude_	*P*_adjusted_	CC (*n* = 791)	CT + TT (*n* = 343)	*P*_crude_	*P*_adjusted_
All vegetables	106.9 ± 74.6	105.3 ± 69.6	0.799	0.853	108.0 ± 73.0	106.7 ± 69.7	0.862	0.835
All fruits	221.1 ± 213.1	227.3 ± 273.0	0.421	0.407	237.3 ± 228.8	204.4 ± 185.9	0.092	0.126
Meats	62.2 ± 43.0	63.5 ± 47.5	0.935	0.919	68.0 ± 43.3	65.0 ± 36.5	0.577	0.501
White meats	8.2 ± 10.0	8.0 ± 9.0	0.535	0.590	8.7 ± 10.2	8.3 ± 8.2	0.484	0.592
Red meats	52.8 ± 48.9	55.8 ± 56.2	0.730	0.718	61.5 ± 53.0	57.4 ± 42.1	0.961	0.907
Organ meats	1.4 ± 3.6	1.4 ± 4.5	0.638	0.656	1.6 ± 3.9	1.6 ± 3.6	0.259	0.333
Fishes	23.7 ± 24.3	23.7 ± 20.7	0.572	0.650	27.5 ± 22.9	28.1 ± 24.5	0.480	0.364
Blue fishes	5.3 ± 6.7	4.6 ± 4.8	0.212	0.185	6.2 ± 7.6	6.0 ± 6.6	0.388	0.666
White fishes	13.9 ± 18.5	15.0 ± 18.1	0.385	0.435	16.4 ± 18.2	16.7 ± 22.0	0.802	0.721
Sweets and snacks	5.2 ± 12.7	4.9 ± 10.1	0.289	0.330	4.3 ± 8.1	6.4 ± 10.5	0.003	0.005
Coffee	3.4 ± 3.6	3.7 ± 3.9	0.225	0.184	4.0 ± 3.9	3.9 ± 3.7	0.564	0.750
Coffee creamer	3.7 ± 4.3	4.0 ± 4.6	0.022	0.017	4.0 ± 4.6	4.1 ± 4.5	0.448	0.495

BMI, body mass index; *n*, number of subjects.

Values are presented as the means and standard deviations.

*P*_crudes_ were from crude general linear models.

*P*_adjusteds_ were from general linear models, including region, age, body mass index, education level, alcohol consumption, smoking status, and physical activity level.

In obese females, T allele carriers tended to consume more organ meats, blue fishes, and coffee than T allele non-carriers (Table 4). In particular, the association between T alleles and food intake differed significantly for coffee creamer intake. T alleles of *FTO* rs1121980 in obese females were significantly associated with the higher intake of coffee creamer, and this significant gene-dietary intake association was retained in statistical model II considering subjects’ lifestyle factors (2.4 ± 3.4 and 1.9 ± 3.1 g/day, *P*_adjusted_ = 0.004). These results suggested that although the type of foods was different by sex and BMI, the presence of the *FTO*-obesity risk allele was associated with some fat-tasting foods.

**Table 4:** Consumption of selected foods (g/day) and the association with the FTO rs1121980 genotype in females

Food group	BMI < 25 (*n* = 1,921, 59.8%)				BMI ≥ 25 (*n* = 1,292, 40.2%)			
	CC (*n* = 1,384)	CT + TT (*n* = 537)	*P*_crude_	*P*_adjusted_	CC (*n* = 902)	CT + TT (*n* = 390)	*P*_crude_	*P*_adjusted_
All vegetables	110.7 ± 87.1	111.4 ± 79.9	0.371	0.437	111.5 ± 79.8	108.4 ± 73.7	0.643	0.673
All fruits	308.2 ± 288.2	309.5 ± 287.3	0.395	0.474	306.3 ± 272.2	294.5 ± 268.9	0.785	0.703
Meats	45.1 ± 40.1	46.3 ± 39.1	0.291	0.279	42.6 ± 38.2	43.5 ± 35.2	0.113	0.162
White meats	7.0 ± 10.6	7.6 ± 11.0	0.079	0.056	7.4 ± 15.4	7.0 ± 9.4	0.188	0.297
Red meats	39.0 ± 48.5	38.4 ± 38.7	0.947	0.959	36.3 ± 44.0	36.3 ± 35.9	0.127	0.173
Organ meats	0.5 ± 2.8	0.5 ± 2.6	0.331	0.313	0.6 ± 2.4	0.8 ± 2.6	0.008	0.012
Fishes	24.0 ± 26.9	22.0 ± 20.6	0.829	0.747	22.8 ± 22.8	23.2 ± 21.5	0.238	0.253
Blue fishes	4.8 ± 6.6	5.0 ± 6.3	0.240	0.248	4.9 ± 6.7	5.5 ± 6.8	0.008	0.009
White fishes	16.8 ± 25.9	15.0 ± 16.8	0.644	0.592	16.4 ± 21.1	16.7 ± 21.3	0.232	0.202
Sweets and snacks	5.4 ± 11.2	5.4 ± 10.3	0.283	0.238	4.1 ± 8.9	4.8 ± 9.2	0.209	0.297
Coffee	2.3 ± 2.8	2.5 ± 3.1	0.650	0.830	2.5 ± 3.0	2.9 ± 3.1	0.010	0.009
Coffee creamer	2.2 ± 3.2	2.1 ± 3.1	0.991	0.938	1.9 ± 3.1	2.4 ± 3.4	0.007	0.004

BMI, body mass index; *n*, number of subjects.

Values are presented as the means and standard deviations.

*P*_crudes_ were from crude general linear models.

*P*_adjusteds_ were from general linear models, including region, age, body mass index, education level, alcohol consumption, smoking status, and physical activity level.

## Discussion

*FTO* is a well-known genetic risk factor for obesity. This study examined the association between the genetic variation rs1121980 in *FTO* and dietary and nutritional intake in Koreans with an epidemiological approach. The findings of the current study suggested that rs1121980 T alleles in obese subjects were associated with some fat-tasting food intake including coffee creamer.

Earlier studies have suggested that variations in *FTO* may be associated with adipose metabolism and the regulation of food preference/intake. A common genetic variation rs9939609 in *FTO* was evident to be associated with BMI in multi-cohort studies (19). Genetic variation was also associated with both nutritional and food group-based intake (7, 8). Individuals with a homozygous genotype for another obesity risk allele in *FTO* rs1121980 were also associated with increased energy intake, especially fat intake (9). One meta-analyze suggested that risk alleles in *FTO* showed a significant association with lower total energy intake, but higher fat intake (20). However, inconsistencies in findings regarding *FTO* genetic variations and obesity and its related factors have also been reported. In another meta-analysis with multiple ethnicities, including African/Hispanic and Caucasian populations, no significant associations were observed between genetic variations and obesity risk (21). In a study in Europeans, rs1121980 was not associated with total energy, carbohydrate, fat, or protein intake (22). One Korean study reported that *FTO* variations were associated with fat intake, but this was only significant in adolescent and children, but not in adults (23). The cause for such differential findings regarding nutritional intake and *FTO* genetic variation in the studies remained unclear (24). However, *FTO* genetic variation studies have been conducted in various ethnicities, ages, sexes, and SNPs. The differences in ethnicity-specific genetics, physiological metabolism, and dietary culture might be associated with such varied findings. In line with this, in the present study, *FTO* genetic variation had no significant effect on macronutrient intake in Koreans. The reasons for the lack of association between the rs1121980 genetic variation and macronutrient intake in this Korean population may be hypothesized as follows: Koreans over 40 years old generally retain traditional eating habits centered on carbohydrates and vegetables. This type of traditional Korean diet contains less fat and sugar (25). The average calorie, fat, and sugar intakes of these Koreans were lower than those of subjects in previous studies, showing a significant association between genetic variation and dietary intake (26, 27). For this reason, the consumption of macronutrients was not decisively evident between genotype groups in Koreans.

In the present study, the *FTO* genotype tended to be associated with certain types of food, taking into account the BMI level and sex, although the effect of genetic variation was minimal in macronutrient intake. This gene-dietary intake association was mainly evident in both males and females with obesity. In females, the food that showed a decisive association with the *FTO* genotype was coffee creamer, which may be linked to fat taste. Coffee creamer, which Koreans usually add to instant coffee, is mainly made from vegetable oils with some food additives, such as emulsifiers, spices, and milk protein (28). In Korea, the fat content of coffee creamer is 7.7–14.0%, of which more than 99% are saturated fatty acids (29). In the case of coffee consumption, Koreans over the age of 40 prefer coffee with sugar or coffee creamer rather than black coffee. According to one Korean study, 50.3% of Koreans over the age of 40 had an instant coffee mix with sugar and cream (creamer) (30). The putative association between coffee preferences and the T allele in obese females may also possibly be attributed to the addition of coffee creamer to instant coffee. In addition, female T allele carriers tended to consume more organ meat and blue fish than poultry and white fish. Those organ meats and blue fish are also higher in fat than lean meat and white fish. Obese males with the T allele tended to consume more snacks and sweets, such as chocolate, cakes, biscuits, and ice cream, than fatty food. However, these types of foods also tend to have both high fat and sugar contents (31). Earlier studies of Swedish MDCS cohorts have reported that the obesity-susceptible minor A allele in *FTO* rs9939609 had no significant association with fat intake depending on genotype. The genetic variants seemed to be associated with the consumption of soft drinks and sucrose (8). However, *FTO* A allele carriers also showed a higher consumption of biscuits, pastries, and high-fat meat than A allele non-carriers (8). Other studies have observed an association between the *FTO* genotype and BMI among subjects consuming a high-fat diet, especially saturated fat and trans-fat (7, 9, 32, 33). Considering these and current findings comprehensively, it could not be dismissed that the genetic variation in *FTO* regulates food preference and intake in relation to fat taste.

*FTO* is known to be the critical locus in obesity etiology, but its detailed pathological role has not yet been fully identified. However, earlier studies have shown that the *FTO* gene increases the risk of obesity in association with BMI and the hormone ghrelin, which promotes appetite (5, 6). In addition to BMI and ghrelin, the results of this study may reveal that *FTO* is associated with appetite control and dietary intake, particularly the consumption of foods with fat taste-energy dense food. Human taste can perceive sweet, sour, salty, bitter, and umami (34). Additionally, fat taste was recently recognized as the sixth taste. Multiple proteins seemed to be involved in fat-sensing mechanisms. Cluster of differentiation 36 is a protein that recognizes the taste of fat (35) and is known to be associated with a mechanism for recognizing long-chain fatty acids in cell membranes and recognizing the taste of fat (36). In addition, some types of G protein-coupled receptors (GPCRs), including GPR40 and GPR120, have been reported to be associated with fatty acid recognition and intake (35). Experimental studies have suggested a role of FTO in protein metabolism and amino acid sensing (37–39). However, these studies were performed in cell lines and animal models and were not confirmed with human sensory tests (37–39). A meta-analysis of the *FTO* obesity risk allele among multiple ethnicities indicated that the risk allele was associated with increased dietary protein intake (40). One Chinese study reported that *FTO* variants were associated with a preference for meat-based meals in children and adolescents (41), although little is known about their relationship with food intake in Asian populations. In the present study, foods rich in protein (e.g. organ meat and blue fish) seemed to be preferred by T-allele carriers. This finding suggests a role for *FTO* in protein sensing and preference. However, the association between coffee creamer preference and genetic variant was clear; moreover, a number of studies have suggested that *FTO* variants are associated with fat and fat-containing energy-dense food preference and intake (23, 42–45). Given these findings, we cannot rule out the idea that *FTO* is associated with a preference for rich and/or high-fat foods. More studies are required to verify the association of *FTO* with nutrient sensing, food preference, and intake in the etiology of obesity.

This study examined the association between *FTO* rs1121980 and dietary intake in Koreans. This study is the first to explore *FTO*-associated food intake in Koreans but could harbor a few limitations. First, this study utilized a cross-sectional design. Since this study collected data only at a certain point in time, it may be difficult to determine the causal relationship between food intake and genetic variants. Second, this study analyzed data for general and dietary intakes for middle- to old-aged adults using data from the KoGES Ansan/Ansung cohort from 2001 to 2002. The KoGES is a representative epidemiological survey in Korea. However, this study may not fully present all dietary and genetic features of Koreans and the recent change in dietary consumption over time. Third, dietary data were collected using the SQFFQ. Korean dishes commonly contain many mixed types of foods and spices. Furthermore, there are limited types of dishes cooked with oils and seasonings. Thus, food-based FFQs may not accurately capture small amounts of food and nutritional intake (46, 47). Finally, alcohol consumption was not included in this study. Alcohol is the most commonly used addictive substance as well as a type of food (48). This study mainly focused on the influence of *FTO* variation on food intake. The degree of alcohol use varies, and there may be more complex linkages between the factors contributing to alcohol consumption. To verify the precise association between *FTO* variation and alcohol consumption, more intensive studies targeting such addictive substances are needed. Therefore, the findings of this study should be interpreted with caution.

## Conclusion

In conclusion, the T allele of rs1121980 in *FTO* is associated with food intake preferences, particularly with preferential intake of rich foods (high in fat; e.g. coffee creamer, blue fish, sweets, and snacks) in Koreans, although these preferences vary according to sex and BMI. Our findings may contribute understanding of the role of *FTO* in dietary intake as well as the etiology of obesity among the Korean population.

## Supplementary Material

Genetic variation rs1121980 in the fat mass and obesity-associated gene (*FTO*) is associated with dietary intake in Koreans

## References

[1] Lee YN, No HG, Lim BS, Kim SH, Lee AR, Kwon SH, et al. Clinical nutrition. New rev. ed. Seoul, Korea: Soohaksa; 2008, pp. 141–148.

[2] Rhee EJ. Prevalence and current management of cardiovascular risk factors in Korean adults based on fact sheets. Endocrinol Metab 2020; 35(1): 85. doi: 10.3803/EnM.2020.35.1.85 PMC7090302 32207267

[3] Oh SW. Obesity and metabolic syndrome in Korea. Diabetes Metab J 2011; 35(6): 561. doi: 10.4093/dmj.2011.35.6.561 22247896 PMC3253964

[4] Avila C, Holloway AC, Hahn MK, Morrison KM, Restivo M, Anglin R, et al. An overview of links between obesity and mental health. Curr Obes Rep 2015; 4(3): 303–10. doi: 10.1007/s13679-015-0164-9 26627487

[5] Loos RJF, Yeo GSH. The bigger picture of *FTO* – the first GWAS-identified obesity gene. Nat Rev Endocrinol 2014; 10(1): 51–61. doi: 10.1038/nrendo.2013.227 24247219 PMC4188449

[6] Karra E, O’Daly OG, Choudhury AI, Yousseif A, Millership S, Neary MT, et al. A link between *FTO*, ghrelin, and impaired brain food-cue responsivity. J Clin Invest 2013; 123(8): 3539–51. doi: 10.1172/JCI44403 23867619 PMC3726147

[7] Sonestedt E, Roos C, Gullberg B, Ericson U, Wirfält E, Orho-Melander M. Fat and carbohydrate intake modify the association between genetic variation in the *FTO* genotype and obesity. Am J Clin Nutr 2009; 90(5): 1418–25. doi: 10.3945/ajcn.2009.27958 19726594

[8] Brunkwall L, Ericson U, Hellstrand S, Gullberg B, Orho-Melander M, Sonestedt E. Genetic variation in the fat mass and obesity-associated gene (*FTO*) in association with food preferences in healthy adults. Food Nutr Res 2013; 57(1): 20028. doi: 10.3402/fnr.v57i0.20028 PMC3625705 23589710

[9] Corella D, Arnett DK, Tucker KL, Kabagambe EK, Tsai M, Parnell LD, et al. A high intake of saturated fatty acids strengthens the association between the fat mass and obesity-associated gene and BMI. J Nutr 2011; 141: 2219–25. doi: 10.3945/jn.111.143826 22049296 PMC3223879

[10] Westenhoefer J. Age and gender dependent profile of food choice. Forum Nutr 2005; 57: 44–51. doi: 10.1159/000083753 15702587

[11] Kim Y, Han BG, KoGES Group. Cohort profile: the Korean genome and epidemiology study (KoGES) consortium. Int J Epidemiol 2017; 46(2): e20. doi: 10.1093/ije/dyv316 27085081 PMC5837648

[12] Min HS, Kim YJ. Quantification of physical activity using epidemiologic questionnaire data in Korea. Public Health Wkly Rep 2012; 5(33): 620–3.

[13] Ahn Y, Kwon E, Shim JE, Park MK, Joo Y, Kimm K, et al. Validation and reproducibility of food frequency questionnaire for Korean genome epidemiologic study. Eur J Clin Nutr 2007; 61(12): 1435–41. doi: 10.1038/sj.ejcn.1602657 17299477

[14] Ministry of Health and Welfare, Korean Nutrition Society. Dietary reference intakes for Koreans. 7th ed. Seoul: Ministry of Health and Welfare; 2000.

[15] Cho YS, Go MJ, Kim YJ, Heo JY, Oh JH, Ban HJ, et al. A large-scale genome-wide association study of Asian populations uncovers genetic factors influencing eight quantitative traits. Nat Genet 2009; 41(5): 527–34. doi: 10.1038/ng.357 19396169

[16] Machiela MJ, Chanock SJ. LDlink: a web-based application for exploring population specific haplotype structure and linking correlated alleles of possible functional variants. Bioinformatics 2015; 31: 3555–7. doi: 10.1093/bioinformatics/btv402 26139635 PMC4626747

[17] Willett WC, Howe GR, Kushi LH. Adjustment for total energy intake in epidemiologic studies. Am J Clin Nutr 1997; 65(4): 1220S–8S. doi: 10.1093/ajcn/65.4.1220S 9094926

[18] Seo MH, Lee WY, Kim SS, Kang JH, Kang JH, Kim KK, et al. 2018 Korean society for the study of obesity guideline for the management of obesity in Korea. J Obes Metab Syndr 2019; 28(1): 40–5. doi: 10.7570/jomes.2019.28.1.40 31089578 PMC6484940

[19] Frayling TM, Timpson NJ, Weedon MN, Zeggini E, Freathy RM, Lindgren CM, et al. A common variant in the *FTO* gene is associated with body mass index and predisposes to childhood and adult obesity. Science 2007; 316(5826): 889–94. doi: 10.1126/science.1141634 17434869 PMC2646098

[20] Livingstone KM, Celis-Morales C, Lara J, Ashor AW, Lovegrove JA, Martinez JA, et al. Associations between *FTO* genotype and total energy and macronutrient intake in adults: a systematic review and meta-analysis. Obes Rev 2015; 16(8): 666–78. doi: 10.1111/obr.12290 26016642

[21] Peng S, Zhu Y, Xu F, Ren X, Li X, Lai M. *FTO* gene polymorphisms and obesity risk: a meta-analysis. BMC Med 2011; 9(1): 1–15. doi: 10.1186/1741-7015-9-71 21651756 PMC3118373

[22] Bauer F, Elbers CC, Adan RA, Loos RJ, Onland-Moret NC, Grobbee DE, et al. Obesity genes identified in genome-wide association studies are associated with adiposity measures and potentially with nutrient-specific food preference. Am J Clin Nutr 2009; 90(4): 951–9. doi: 10.3945/ajcn.2009.27781 19692490

[23] Lee HJ, Kim IK, Kang JH, Ahn Y, Han BG, Lee JY, et al. Effects of common *FTO* gene variants associated with BMI on dietary intake and physical activity in Koreans. Clin Chim Acta 2010; 411(21–22): 1716–22. doi: 10.1016/j.cca.2010.07.010 20650268

[24] Drabsch T, Gatzemeier J, Pfadenhauer L, Hauner H, Holzapfel C. Associations between single nucleotide polymorphisms and total energy, carbohydrate, and fat intakes: a systematic review. Adv Nutr 2018; 9(4): 425–53. doi: 10.1093/advances/nmy024 30032228 PMC6054249

[25] Lee CH, Joo YJ, Ahn KO, Ryu SS. The changes in the dietary pattern and health and nutritional status of Korean during the last one century. Kor J Dietary Cult 1988; 3(4): 397–406.

[26] Song Y, Joung H. A traditional Korean dietary pattern and metabolic syndrome abnormalities. Nutr Metab Cardiovasc Dis 2012; 22(5): 456–62. doi: 10.1016/j.numecd.2010.09.002 21215606

[27] Kuczmarski MF, Bodt BA, Shupe ES, Zonderman AB, Evans MK. Dietary patterns associated with lower 10-year atherosclerotic cardiovascular disease risk among urban African-American and white adults consuming western diets. Nutrients 2018; 10(2): 158. doi: 10.3390/nu10020158 29385036 PMC5852734

[28] Knightly WH. The role of ingredients in the formulation of coffee whiteners. J Food Technol 1969; 23: 171–73, 180, 182.

[29] Lee BE, Lee HJ, Cho EA, Hwang KT. Fatty acid compositions of fats in commercial coffee creamers and instant coffee mixes and their sensory characteristics. J Kor Soc Food Sci Nutr 2012; 41(3): 362–8. doi: 10.3746/jkfn.2012.41.3.362

[30] Kim AN, Youn JY, Cho HJ, Jin T, Shin SA, Lee JE. Comparison of 24-hour recalls with a food frequency questionnaire in assessing coffee consumption: the health examinees (HEXA) study. Kor J Community Nutr 2020; 25(1): 48–60. doi: 10.5720/kjcn.2020.25.1.48

[31] Iatridi V, Hayes JE, Yeomans MR. Reconsidering the classification of sweet taste liker phenotypes: a methodological review. Food Qual Prefer 2019; 72: 56–76. doi: 10.1016/j.foodqual.2018.09.001

[32] Phillips CM, Kesse-Guyot E, McManus R, Hercberg S, Lairon D, Planells R, et al. High dietary saturated fat intake accentuates obesity risk associated with the fat mass and obesity associated gene in adults. J Nutr 2012; 142: 824–31. doi: 10.3945/jn.111.153460 22457394

[33] Koochakpour G, Esfandiar Z, Hosseini-Esfahani F, Mirmiran P, Daneshpour MS, Sedaghati-Khayat B, et al. Evaluating the interaction of common *FTO* genetic variants, added sugar, and trans-fatty acid intakes in altering obesity phenotypes. Nutr Metab Cardiovas 2019; 29(5): 474–80. doi: 10.1016/j.numecd.2019.01.005 30954417

[34] Breslin PA. An evolutionary perspective on food and human taste. Curr Biol 2013; 23(9): R409–18. doi: 10.1016/j.cub.2013.04.010 23660364 PMC3680351

[35] Liu D, Archer N, Duesing K, Hannan G, Keast R. Mechanism of fat taste perception: association with diet and obesity. Prog Lipid Res 2016; 63: 41–9. doi: 10.1016/j.plipres.2016.03.002 27155595

[36] Febbraio M, Hajjar DP, Silverstein RL. CD36: a class B scavenger receptor involved in angiogenesis, atherosclerosis, inflammation, and lipid metabolism. J Clin Invest 2001; 108(6): 785–91. doi: 10.1172/JCI14006 11560944 PMC200943

[37] Ma M, Harding HP, O’Rahilly S, Ron D, Yeo GS. Kinetic analysis of FTO (fat mass and obesity-associated) reveals that it is unlikely to function as a sensor for 2-oxoglutarate. Biochem J 2012; 444(2): 183–7. doi: 10.1042/bj20120065 22435707 PMC7617487

[38] Cheung MK, Gulati P, O’Rahilly S, Yeo GS. FTO expression is regulated by availability of essential amino acids. Int J Obes 2013; 37(5): 744–7. doi: 10.1038/ijo.2012.77 22614055

[39] McMurray F, Church CD, Larder R, Nicholson G, Wells S, Teboul L, et al. Adult onset global loss of the *FTO* gene alters body composition and metabolism in the mouse. PLoS Genet 2013; 9(1): e1003166. doi: 10.1371/journal.pgen.1003166 23300482 PMC3536712

[40] Qi Q, Kilpeläinen TO, Downer MK, Tanaka T, Smith CE, Sluijs I, et al. *FTO* genetic variants, dietary intake and body mass index: insights from 177,330 individuals. Hum Mol Genet 2014; 23(25): 6961–72. doi: 10.1093/hmg/ddu411 25104851 PMC4271061

[41] Yang M, Xu Y, Liang L, Fu J, Xiong F, Liu G, et al. The effects of genetic variation in *FTO* rs9939609 on obesity and dietary preferences in Chinese Han children and adolescents. PLoS One 2014; 9(8): e104574. doi: 10.1371/journal.pone.0104574 25110886 PMC4128666

[42] Mehrdad M, Doaei S, Gholamalizadeh M, Eftekhari MH. The association between *FTO* genotype with macronutrients and calorie intake in overweight adults. Lipids Health Dis 2020; 19(1): 197. doi: 10.1186/s12944-020-01372-x 32843047 PMC7449073

[43] Melhorn SJ, Askren MK, Chung WK, Kratz M, Bosch TA, Tyagi V, et al. *FTO* genotype impacts food intake and corticolimbic activation. Am J Clin Nutr 2018; 107(2): 145–54. doi: 10.1093/ajcn/nqx029 29529147 PMC6454473

[44] Daya M, Pujianto DA, Witjaksono F, Priliani L, Susanto J, Lukito W, et al. Obesity risk and preference for high dietary fat intake are determined by *FTO* rs9939609 gene polymorphism in selected Indonesian adults. Asia Pac J Clin Nutr 2019; 28(1): 183–91. doi: 10.6133/apjcn.201903_28(1).0024 30896430

[45] Harbron J, van der Merwe L, Zaahl MG, Kotze MJ, Senekal M. Fat mass and obesity-associated (*FTO*) gene polymorphisms are associated with physical activity, food intake, eating behaviors, psychological health, and modeled change in body mass index in overweight/obese Caucasian adults. Nutrients 2014; 6(8): 3130–52. doi: 10.3390/nu6083130 25102252 PMC4145299

[46] Yun SH, Choi BY, Kim MK. The effect of seasoning on the distribution of nutrient intakes by a food-frequency questionnaire in a rural area. Kor J Nutr 2009; 42(3): 246–55. doi: 10.4163/kjn.2009.42.3.246

[47] Shim JS, Oh K, Kim HC. Dietary assessment methods in epidemiologic studies. Epidemiol Health 2014; 36: e2014009. doi: 10.4178/epih/e2014009 25078382 PMC4154347

[48] Nutt D, King LA, Saulsbury W, Blakemore C. Development of a rational scale to assess the harm of drugs of potential misuse. Lancet 2007; 369(9566): 1047–53. doi: 10.1016/S0140-6736(07)60464-4 17382831

